# Control-Oriented Modelling of a 3D-Printed Soft Actuator

**DOI:** 10.3390/ma12010071

**Published:** 2018-12-26

**Authors:** Ali Zolfagharian, Akif Kaynak, Sui Yang Khoo, Jun Zhang, Saeid Nahavandi, Abbas Kouzani

**Affiliations:** 1 School of Engineering, Deakin University, 3216 Geelong, Australia; sui.khoo@deakin.edu.au (S.Y.K.); tzy@deakin.edu.au (J.Z.); 2 Institute for Intelligent Systems Research and Innovation (IISRI), Deakin University, 3216 Geelong, Australia; saeid.nahavandi@deakin.edu

**Keywords:** modeling, soft actuator, soft robot, 3D print

## Abstract

A new type of soft actuator was developed by using hydrogel materials and three-dimensional (3D) printing technology, attracting the attention of researchers in the soft robotics field. Due to parametric uncertainties of such actuators, which originate in both a custom design nature of 3D printing as well as time and voltage variant characteristics of polyelectrolyte actuators, a sophisticated model to estimate their behaviour is required. This paper presents a practical modeling approach for the deflection of a 3D printed soft actuator. The suggested model is composed of electrical and mechanical dynamic models while the earlier version describes the actuator as a resistive-capacitive (RC) circuit. The latter model relates the ionic charges to the bending of an actuator. The experimental results were acquired to estimate the transfer function parameters of the developed model incorporating Takagi-Sugeno (T-S) fuzzy sets. The proposed model was successful in estimating the end-point trajectory of the actuator, especially in response to a broad range of input voltage variation. With some modifications in the electromechanical aspects of the model, the proposed modelling method can be used with other 3D printed soft actuators.

## 1. Introduction

In contrast to building robots from rigid materials in conventional robotics, soft, responsive, flexible, and compliant materials have been used to make composite materials that can mimic biological systems. The study of such responsive composites is often referred to as soft robotics, which is a specific category of the field of robotics. There has been a growing interest in these materials, which have potential applications in sensors and actuators. Functional components composed of soft and active materials can be 3D-printed. Furthermore, it was observed that monolithic structures independent of pneumatics or fluidics systems, which have their own shortcomings [1], can be developed as muscle-like actuators by means of materials such as shape memory alloys, shape memory polymers, or responsive hydrogel materials [1,2,3].

Using 3D bio-printing as part of the fabrication of soft actuators from responsive materials such as hydrogel actuators with multi-material compositions that have spatial control was recently investigated [4]. In a recent study, polyelectrolyte chitosan hydrogel was used in the 3D-printing of a soft actuator [5]. Chitosan was found to be an appropriate material for drug release, cell manipulation, and similar biological applications where the actuation and responsiveness to external stimuli is essential [6]. This is mainly referred to its antibacterial properties, which are attributed to free amino groups on the hydrogel backbone.

From one point of view, hydrogels can be divided into two groups in terms of responsive behaviour to the potential gradient. A group known as non-ionic hydrogels do not demonstrate a notable actuation response to input voltage when immersed in electrolyte solutions due to even distribution of hydrated ions on two sides of hydrogels. Polyelectrolyte hydrogels, however, react differently from non-ionic hydrogels when immersed into electrolyte solutions. Upon immersion in electrolyte solutions and applying voltages, polyelectrolyte hydrogels show actuation behaviour based on parameters like strength and polarity of input voltages as well as the pH of solutions [7]. Various key factors, such as ionic charge and crosslinking densities as well as polymer and external electrolyte concentrations [7], have been identified to affect the amount of bending of polyelectrolyte hydrogels in response to an input voltage. For chitosan hydrogel, when is immersed into an electrolyte solution with a high pH, the carboxylic group would be deprotonated in order for the hydrogel actuator to take a negative charge. Then, upon the application of voltage potentials on electrodes, the hydrated cations enter to one side of the actuator more than the other side. This result in an ionic strength difference between inside and outside of the hydrogel leads to a greater osmotic pressure on one side of the hydrogel than the other side, which, in turn, leads to a bending of the hydrogel actuator to the counter electrode or cathode. Thus, the gel near the anode swells and causes it to bend toward the cathode [8]. Along with the osmotic pressure gradient, the ionic strength also affects the extent of bending of the polyelectrolyte soft actuator. Due to a higher electric voltage, the movements of ions are accelerated, which lead to faster ionic and osmotic pressure gradients and, therefore, increase both the bending angle and the bending rate of the actuator. Yet, the relation of bending behaviour of the actuator is not always proportional to ionic strength of electrolyte solution because of the shielding effect phenomenon [9]. 

Polyelectrolyte soft actuators have been considered as electro-chemo-mechanical systems that make their modelling quite complicated. The bending and actuation performances of polyelectrolyte soft actuators are influenced by uncertainties and time-varying parameters stemming from back relaxation and modulus variation phenomena. These undesirable factors should be dealt with for further fabrication and applications of such soft actuators. However, with the incorporation of 3D-printing in manufacturing such soft actuators, developing a control-oriented model for estimating the behaviour of these 3D-printed polyelectrolyte soft actuators in real world applications is in high demand.

Studies on dynamics modelling of conventional polyelectrolyte soft actuators have been conducted. Black-box models were used in some works for calculating the curvature of the actuator based on input voltage but were not scalable and too simple to describe the actuator performance entirely [10]. Advanced grey-box models based on electrical circuit models, like RC [11] and distributed transmission line models [12], have also been developed to correlate the applied voltage to the bending of the actuators [13]. More complex white-box models [10,11,12] considering complicated electro-chemo-mechanical principals were developed to explain the more insightful details of underlying physics for accurate dynamics modelling of the polyelectrolyte actuators. Yet, these models were too complex and not appropriate for real time control application of such actuators. This study establishes a mathematical grey-box relation of the 3D-printed polyelectrolyte soft actuator by coupling both mechanical and electrical dynamics of the actuators.

Takagi-Sugeno (T-S) fuzzy modelling has been a practical approach in control designs applications [14,15]. This study develops a reliable model of the 3D-printed polyelectrolyte soft actuator based on the T-S fuzzy modelling strategy. The proposed model relates the different input voltages applied to the actuator to the bending of the actuator via a universal T-S model surfing among the voltage dependent sub-models. This provides a scalable and practical model for further control applications of such systems.

The rest of the paper is comprised of the following sections. First, 3D printing of the polyelectrolyte soft actuator using chitosan is explained. Then, an electro-chemo-mechanical model of the 3D-printed polyelectrolyte actuator is developed. Lastly, the developed model is validated via the experimental test data.

## 2. Fabrication of the Polyelectrolyte Actuator

**Figure 1:**
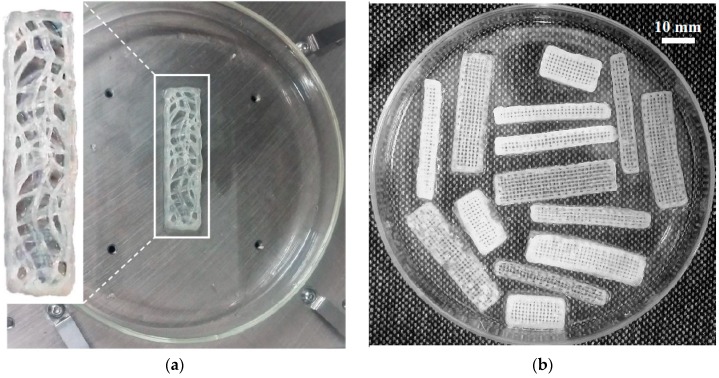
(**a**) An arbitrary pattern 3D printed polyelectrolyte actuator and (**b**) different patterns and sizes of 3D-printed polyelectrolyte actuators.

Like other 3D-printed products, first, the actuator model was drawn in Solidworks 2016 (Dassault Systemes, Waltham, MA, USA) and then the model was imported into an EnvisionTEC GmbH Bioplotter V2.2 software program (EnvisionTEC, Gladbeck, Germany). The required materials including medium molecular weight chitosan (with 75–85% deacetylation degree) and acetic acid solution were purchased from SigmaAldrich (Sydney, NSW, Australia). A mixture of 1.6 g chitosan in 0.8 mL acetic acid solution (1 v/v%) was made under vigorous stirring at 50 °C for 2 h. The resultant 3D-print ink was then prepared through sonication and centrifugation. Lastly, the ready-to-print ink was poured into a low-temperature 3D Bioplotter syringe. For solidifying each layer after extrusion, a solution of Ethanolic Sodium Hydroxide (EtOH-NaOH) with 0.25 M NaOH (Sigma Aldrich), 70 v/v% EtOH (3:7 ratio), was prepared. The porous chitosan beam with the size of 40 mm by 8 mm by 2 mm was printed layer-by-layer (Figure 1a). The 3D printing was performed with optimized parameters of the 3D Bioplotter, as explained extensively in an earlier work by the authors [16].

## 3. Electro-Chemo-Mechanical Model of the 3D-Printed Polyelectrolyte Actuator

To circumvent the complexity of multiphysics modeling, this study suggests a scalable model of the polyelectrolyte actuator. Doing so, various factors are considered in the behavior of the actuators to estimate the dynamics parameters more realistically. Thus, the actuator model comprises both electrochemical and electromechanical models considering their dynamics coupling.

### 3.1. Electrochemical Modelling 

**Figure 2:**
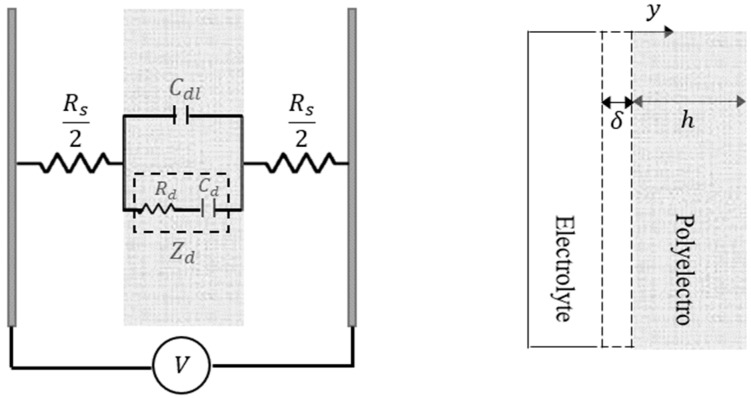
Equivalent RC circuit where $Z_{d}$ is the diffusion impedance, $C_{dl}$ denotes the double layer capacitance, and $R_{s}$ refers to the resistance of the electrolyte solution.

Figure 2 shows an electrochemical RC model that gives the relation between applied voltage and current flow across the polyelectrolyte gel. A diffusion model to represent the current flow across the 3D-printed polyelectrolyte actuator is calculated from the Fick’s law of diffusion as:

(1) $$i_{D}\left(t\right)=-F\times A\times D\times \frac{\partial c\left(y,t\right)}{\partial y},$$

where *c* refers to the ion concentration, *y* indicates the path along the thickness of the actuator, *D* is the diffusion coefficient constant, *A* is the area between the interface of electrolyte solution and polyelectrolyte actuator, and *F* denotes the Faraday constant. Then, the current running across the double layer capacitance can be calculated assuming the double-layer capacitance thickness as δ:

(2) $$i_{C}\left(t\right)=F\times A\times \delta \times \frac{\partial c\left(y,t\right)}{\partial y}.$$

Next, for a 3D-printed polyelectrolyte actuator with the thickness of the *h*, the diffusion equation model can be written as:

(3) $$\frac{\partial c\left(y,t\right)}{\partial t}=D\frac{\partial^{2}c\left(y,t\right)}{\partial y^{2}}\;0<y<h,\;\frac{\partial c\left(y,t\right)}{\partial t}\left(@y=h\right)=0.$$

Lastly, an electrochemical model relating the current flow across the actuator to the applied voltage can be obtained by simultaneously solving Equations (1)–(3) as:

(4) $$\frac{I\left(s\right)}{V\left(s\right)}=\frac{s\left[\frac{\sqrt{D}}{\delta }\tanh\left(h\sqrt{\frac{s}{D}}\right)+\sqrt{s}\right]}{\frac{\sqrt{s}}{C_{dl}}+s\sqrt{s}R_{s}+R_{s}\frac{\sqrt{D}}{\delta }s\tanh\left(h\sqrt{\frac{s}{D}}\right)}.$$

### 3.2. Electromechanical Modelling 

An electromechanical model that relates the osmotic pressure caused by input voltage to the bending of 3D-printed polyelectrolyte actuator is developed in this section. Assuming the strain-to-charge ratio α, the relation between the charges density $\rho_{ch}$ and the in-plane strain ε in polyelectrolyte actuators can be written as [17,18]:

(5) $$\varepsilon =\alpha \rho_{ch},$$

where, for a 3D-printed soft actuator with a size of *W* and *L* as width and length, respectively, $\rho_{ch}$ can be calculated as:

(6) $$\rho_{ch}=\frac{1}{WLh}\int_{0}^{t}I\left(t\right)dt\overset{L}{\to }\rho_{ch}\left(s\right)=\frac{I\left(s\right)}{sWLh},$$

**Figure 3:**
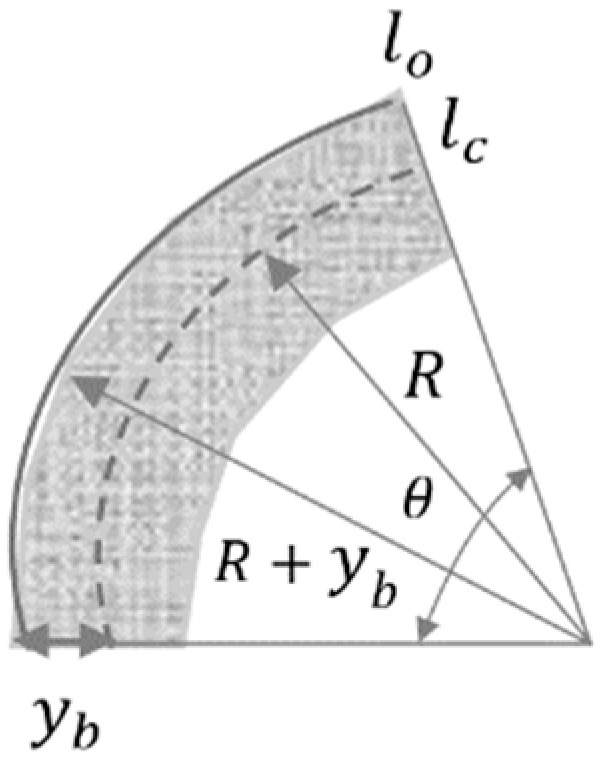
Schematic of the bending of the printed actuator.

Generally, actuators strains are calculated knowing the tip displacement as well as the initial length and thickness of the actuators when the bending curvature is comparatively small. However, the curvature radius is not negligible when the soft actuators experience larger bending. In this study, as shown in Figure 3 and Equation (7), the strain ε resulted purely from the bending of the actuator, which is obtained as:

(7) $$\varepsilon =\frac{l_{o}-l_{c}}{l_{c}}=\frac{\left(R+y\right)\theta -R\theta }{R\theta }=\frac{y_{b}}{R}=Ky_{b},$$

where $l_{o}$ and $l_{c}$ are the swelled length and the centered length of actuators, θ and R refer to the bending angle and the radius of curvature of the actuator, respectively, $y_{b}$ shows the distance between the outer edge of the swelled layer and the reference plane, and K is the bending curvature of the actuator. To estimate an accurate value of the stress induced on the bending of the actuator, the superposition of the actuations effect and the bending strain are considered together. Therefore, the total induced stress, assuming the Young’s modulus of the 3D printed polyelectrolyte actuator as E, can be calculated as follows.

(8) $$\sigma =E\left(K\pm \alpha \rho_{ch}\right),$$

where the opposite signs represent the expansion and contraction of the actuator sides.

Making the total moments and forces on the actuators equal zero, the bending curvature K can be realized. This is somewhat related to a new defined coefficient *φ* as follows.

(9) $$\sum F=\int_{-h/2}^{0}E\left(Ky_{b}+\alpha \rho_{ch}\right)y_{b}dy_{b}+\int_{0}^{h/2}E\left(Ky_{b}-\alpha \rho_{ch}\right)y_{b}dy_{b}=0,\;\sum \sigma =\int_{-h/2}^{0}E\left(Ky_{b}+\alpha \rho_{ch}\right)dy_{b}+\int_{0}^{h/2}E\left(Ky_{b}-\alpha \rho_{ch}\right)dy_{b}=0,\;K=\varphi \rho_{ch},\;where\;\varphi =\frac{3\alpha }{Eh}.$$

Combining Equations (9) and (6) into Equation (4), the bending curvature can be expressed as:

(10) $$K\left(s\right)=\frac{\varphi V\left(s\right)}{WLh}\frac{\left[\frac{\sqrt{D}}{\delta }\tanh\left(h\sqrt{\frac{s}{D}}\right)+\sqrt{s}\right]}{\frac{\sqrt{s}}{C_{dl}}+R_{s}s\sqrt{s}+R_{s}\frac{\sqrt{D}}{\delta }s\tanh\left(h\sqrt{\frac{s}{D}}\right)}.$$

Lastly, the model relating the applied voltage to the bending of the 3D-printed polyelectrolyte actuator can be obtained by incorporating Equation (6) and Equations (5)–(10) in the following equation [19].

(11) $$\frac{Y\left(s\right)}{V\left(s\right)}=\frac{\varphi }{WLh}\frac{1}{sR_{s}+\frac{1}{C_{dl}\left[1+\frac{\sqrt{D}}{\delta \sqrt{s}}\tanh\left(h\sqrt{\frac{s}{D}}\right)\right]}},\;\mathrm{where},\;Y\left(s\right)=\frac{1}{K\left(s\right)}\pm \sqrt{\frac{1}{K{\left(s\right)}^{2}}-L^{2}}.$$

Equation (11) needs to be restructured to be practical for control applications due to the presence of a hyperbolic tangent term in its denominator, which is dimensionally infinite. To deal with this, the dimensionally infinite hyperbolic tangent term can be replaced by Mittag Leffler’s expansion as:

(12) $$\frac{\tan h\left(\frac{1}{2}\sqrt{\frac{X}{Y}}\right)}{4\sqrt{XY}}=\overset{\infty }{\underset{n=0}{\sum }}\frac{1}{X+n^{2}\left({\left(2n+1\right)}^{2}Y\right)},$$

where X=s and $Y=D/4h^{2}$. This simplification confines the model in a bounded range of frequencies for input voltages within the low interest frequency range for the 3D-printed polyelectrolyte soft actuator. Hence, Equation (12) represents the model for the small values of n, so a fourth order model approximates bending displacement of the 3D-printed soft actuator as:

(13) $$\frac{Y\left(s\right)}{V\left(s\right)}=e^{\beta s}\frac{\left(s+z_{1}\right)\left(s+z_{2}\right)\left(s+z_{3}\right)}{\left(s+p_{1}\right)\left(s+p_{2}\right)\left(s+p_{3}\right)\left(s+p_{4}\right)},$$

where β is a real value and z_i_’s (*n = 1*, *2*, *3*) are the zeros and p_i_’s (*n = 1*, *2*, *3*, *4*) are the poles of the soft actuator transfer function.

### 3.3. T-S Fuzzy System Modeling Formulation

Consider a simplified dynamic system without uncertainty systems as:

(14) $$\dot{x}=A\left(x\right)+B\left(x\right)u.$$

Fuzzy inference rules for the T-S fuzzy dynamic model of Equation 16 can be described as follows [20].

$R^{i}:IF\;z_{1}is\;w_{1}^{i}\;AND\ldots \;z_{n}\;is\;w_{n}^{i}\;$

THEN

(15) $$\dot{x}=A^{i}\left(x\right)+B^{i}\left(x\right)u\;\mathrm{for}\;i=1,\ldots ,\;m,$$

where the matrices $A^{i}\in R^{n\times n}$ and $B^{i}\in R^{n\times 1}$ represent the subsystem parameters, $z={\left(z_{1},\ldots ,z_{n}\right)}^{T}$, behaviour. x is the system state variable vector and u is the system input. $R^{i}$ represents the $i^{th}$ fuzzy inference rule, $z\left(t\right)=\left[z_{1}\left(t\right)\;z_{2}\left(t\right)\ldots \;z_{n}\left(t\right)\right]$ among m number of inference rules.

Knowing $M_{j}\left(j=1,\ldots ,n\right)$ are the fuzzy sets, the inferred fuzzy set $w^{i}$ can be used to calculate $\mu_{i}$ as the normalized fuzzy membership function of inferred fuzzy sets as follows.

(16) $$w^{i}=\overset{n}{\underset{j=1}{\prod }}M_{j}^{i},\;\mu^{i}=\frac{w^{i}}{\sum_{i=1}^{m}w^{i}},\;and\;\sum_{i=1}^{m}\mu^{i}=1.$$

The singleton fuzzifier, product inference, and center-average defuzzifier are used to form the global dynamic fuzzy model of the nominal system of Equation (17) as:

(17) $$\dot{x}=A\left(\mu \right)x+B\left(\mu \right)u\left(t\right),\;A\left(\mu \right)=\sum_{i=1}^{m}\mu^{i}A^{i},\;B\left(\mu \right)=\sum_{i=1}^{m}\mu^{i}B^{i},\;where\;\mu =\left(\mu^{1},\mu^{2},\ldots ,\mu^{m}\right).$$

### 3.4. Actuator Characterization

**Figure 4:**
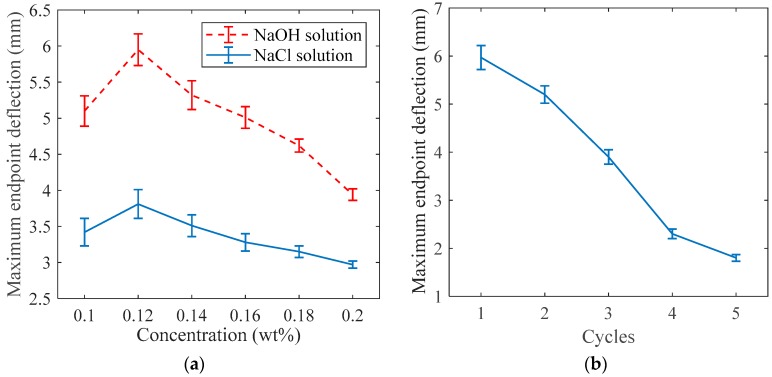
(**a**) Response of the actuators with the same pattern and dimensions to different electrolyte solutions. (**b**) Maximum endpoint deflections of the actuators to 5 V input signal over different cycles. Error bars indicate the standard deviation of the maximum distance measured over the three trials.

The bending of the 3D-printed polyelectrolyte hydrogel actuator can be justified by the Donnan effect phenomenon. This means that the motion of counter-ions initiated by applied voltage leads to the ionic gradient within the hydrogel networks along the direction of the electric field. This results in an osmotic pressure difference within the hydrogel structure, and, consequently, causes the deflection of a 3D-printed actuator toward the counter electrode. Several factors can be considered to characterize the behaviour of the actuator. First, the effects of the electrolyte solution and its concentration on the bending behaviour of the 3D-printed polyelectrolyte actuator should be defined. Doing so, two different electrolyte solutions, NaOH and NaCl, were used to test the 3D-printed actuator endpoint deflection behaviour. The maximum endpoint deflection for the same sizes (40 mm × 8 mm × 2 mm) and patterns of 3D-printed actuators are measured with respect to the ionic strength of the electrolyte solutions. A constant voltage of 5 V was applied between the electrodes. The concentration of NaOH and NaCl solutions ranged from 0.1 to 0.2 M with an increment of 0.2 M for each experiment. The experiments repeated three times and the results are depicted in Figure 4a. Regardless of the concentration of electrolyte solution, the actuator reached a higher deflection in the NaOH solution than in the NaCl solution. Additionally, it is observed that, for both electrolyte solutions, there were an optimum value for electrolyte ionic strength to achieve a maximum bending angle. As shown in Figure 4, this optimum value for the 3D-printed actuator tested here was near 0.12 M. From 0.1 to 0.12 M, the endpoint deflection of the 3D-printed actuator increased. This can be attributed to an increase of the free ions moving from the surrounding solution toward their counter-electrodes. However, if the concentration of the solutions were greater than the critical concentration, 0.12 M, the shielding effect of the poly-ions leads to a reduction in the electrostatic repulsion of the poly-ions, and the subsequent decrease in the endpoint deflection. Hence, in the rest of the paper, all tests are performed in NaOH electrolyte solution of 0.12 M.

Furthermore, several different patterns and dimensions of the actuators with the same material are 3D-printed (Figure 1b). Then, the behaviour of the 3D-printed actuators based on different dimensions are investigated and the results are inserted in Table 1. The results reveal that the maximum endpoint deflection of the actuators increase in proportion to their length. It also shows almost no correlation with the width of the actuator. In addition, the bending deflection was inversely correlated to the thickness of the actuator [4].

To reveal hysteresis behaviour of a 3D-printed actuator, the repeatability tests were performed. The same pattern and size of 3D-printed samples of actuators were excited with a square wave electrical stimulus. The magnitudes of the square waves were selected as 5 V with the period of 220 s and the duty cycle of 50%. The duration of the experiment was set to be 5 times the period, and the performances of the actuators were compared based on change in maximum magnitudes in each cycle, as shown in Figure 4b. The results showed that all actuators reached their maximum deflections at the first set of experiments for a specific excitation voltage.

**Figure 5:**
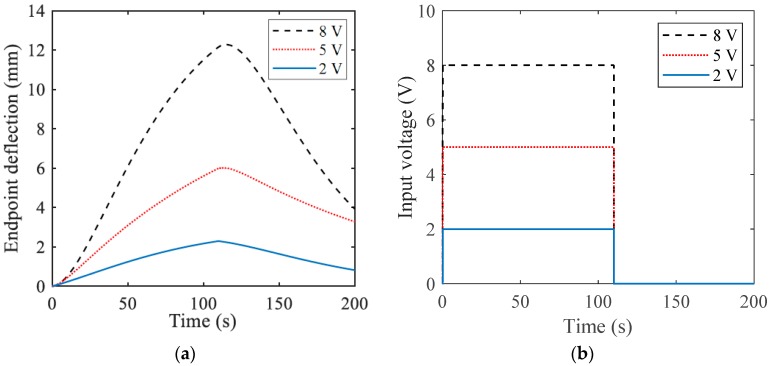
(**a**) Response of the actuators with the same pattern and dimensions to (**b**) different input voltages’ magnitudes. The results are averaged over the three trials.

In the next stage, the actuator’s endpoint deflections were tested over time based on different input voltage magnitudes, as illustrated in Figure 5. From the results, it can be deduced that the actuation performance increased in the higher voltage. However, the occurrence of electrolyses and bubbling in electrolyte caused undesirable effects and limits the application of higher voltages in such actuators.

**Figure 6:**
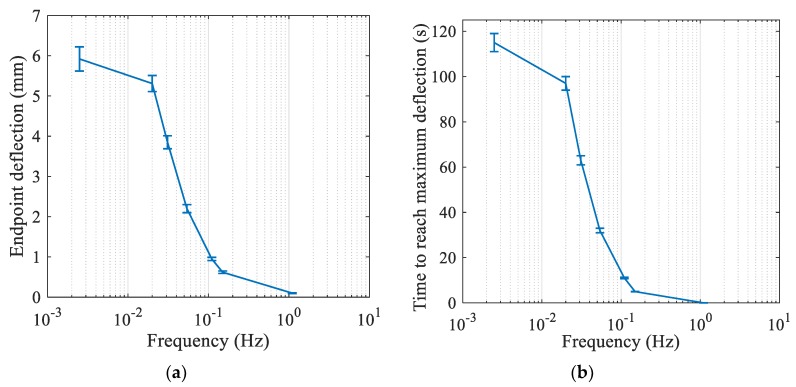
(**a**) Response of actuator’s maximum endpoint deflection to various frequencies under 5 V square waves. (**b**) Response time to first peak. Error bars indicate the standard deviation of the maximum distance measured over the three trials.

The actuator responses under various frequencies were also investigated. The square voltage of 5 V was applied between two electrodes in various frequencies including 0.0025, 0.02, 0.031, 0.054, 0.11, 0.15, and 1.1 Hz. It can be seen from Figure 6a that the maximum endpoint deflections decrease with increasing frequency since the actuators have less time to respond. In addition, response time to the first peak was measured and the results are shown in Figure 6b. From the figure, it can be observed that the actuator reaches the first peak faster as the magnitude of the peaks decreases in higher frequencies.

## 4. Modelling and Experimental Results

The Golubev method [21] is used to estimate an appropriate model for control of the actuator [22,23,24,25]. Using the Golubev method for the input signal, the uncertain transfer function relating the applied voltage to the bending angle of the actuator is estimated as:

(18) $$G\left(s\right)=e^{\beta s}\frac{a_{1}s^{3}+a_{2}s^{2}+a_{3}s+a_{4}}{b_{1}s^{4}+b_{2}s^{3}+b_{3}s^{2}+b_{4}s+b_{5}},$$

**Figure 7:**
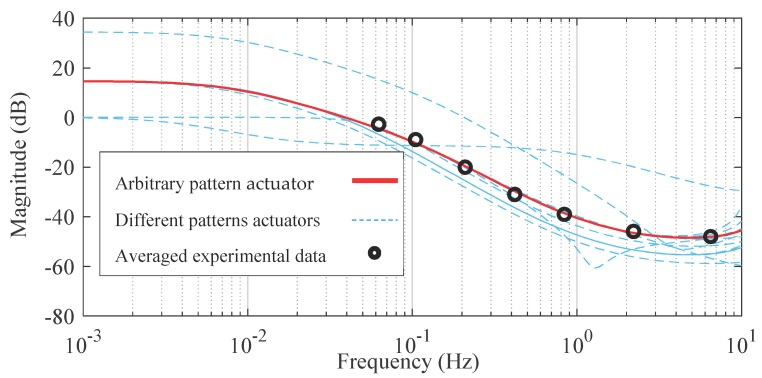
Frequency response system identification of different 3D-printed actuators in response to the 5V input signal.

The frequency response model of the actuators with different patterns and sizes, shown in Figure 1b, is identified based on Equation (18) and the experimental data are depicted in Figure 7. It is observed that changing various parameters of the actuator, like different sizes and patterns, lead to a new set of linear voltage dependent transfer functions. Therefore, for each specific actuator, a system identification of frequency response to different voltage levels is required. Then, the T-S fuzzy model should be incorporated to interrelate the linear transfer functions at different voltage levels for improving the model accuracy.

**Figure 8:**
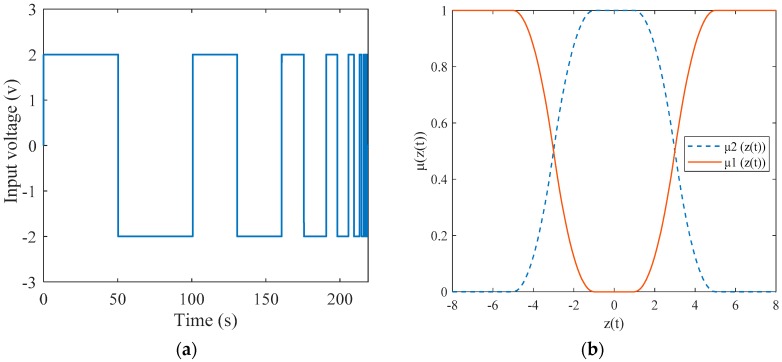
(**a**) An example of input signal for model identification (2 V). (**b**) Membership function of T-S model.

To identify transfer function parameters for the arbitrary pattern actuator S1, lower and upper limit input signals, 2 V (shown in Figure 8a) and 8 V are applied to the system and the range of parameters identified are as follows.

$a_{1}\in \left[0.0063,0.0085\right];a_{2}\in \left[0.6381,0.842\right];a_{3}\in \left[1.152,1.924\right];a_{4}\in \left[1.14,1.97\right];$

$b_{1}\in \left[1,1\right];b_{2}\in \left[0.3631,0.5898\right];b_{3}\in \left[225.5,367.2\right];b_{4}\in \left[14.98,29.41\right];b_{5}\in \left[0.2142,0.2275\right];$

β=−3.67.

Then, a two-rule based T-S model is defined as:

Rule 1: IF |z(t)|≤5 THEN $\dot{x}=A_{1}x\left(t\right)+B_{1}u\left(t\right)$.

Rule 2: IF 5<|z(t)|≤8 THEN $\dot{x}=A_{2}x\left(t\right)+B_{2}u\left(t\right)$.

Lastly, the outputs of the T-S fuzzy model (shown in Figure 7b) can be calculated as:

(19) $$\dot{x}=\mu_{1}\left(z\left(t\right)\right)\left(A_{1}x\left(t\right)+B_{1}u\left(t\right)\right)+\mu_{2}\left(z\left(t\right)\right)\left(A_{2}x\left(t\right)+B_{2}u\left(t\right)\right),\;\mathrm{where}\;\mu_{1}\left(z\left(t\right)\right)+\mu_{2}\left(z\left(t\right)\right)=1.$$

**Figure 9:**
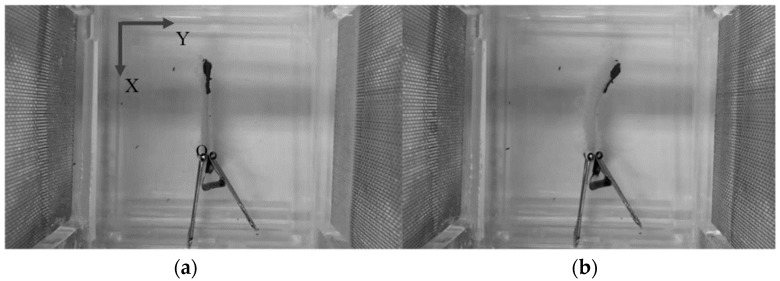
Bending of the printed actuator (**a**) before applying voltage; (**b**) under applied voltage.

**Figure 10:**
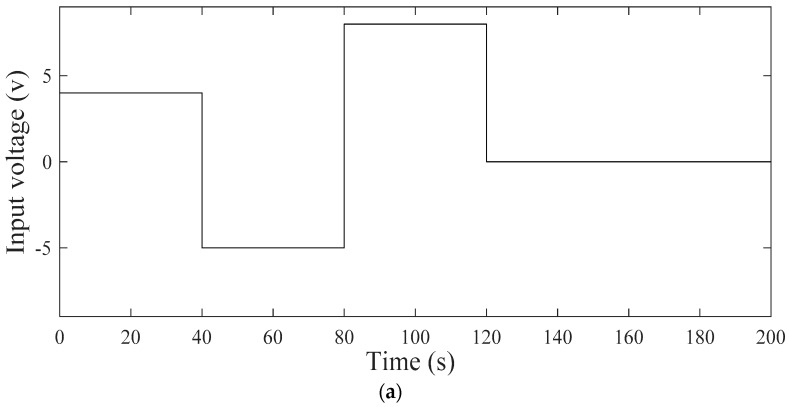

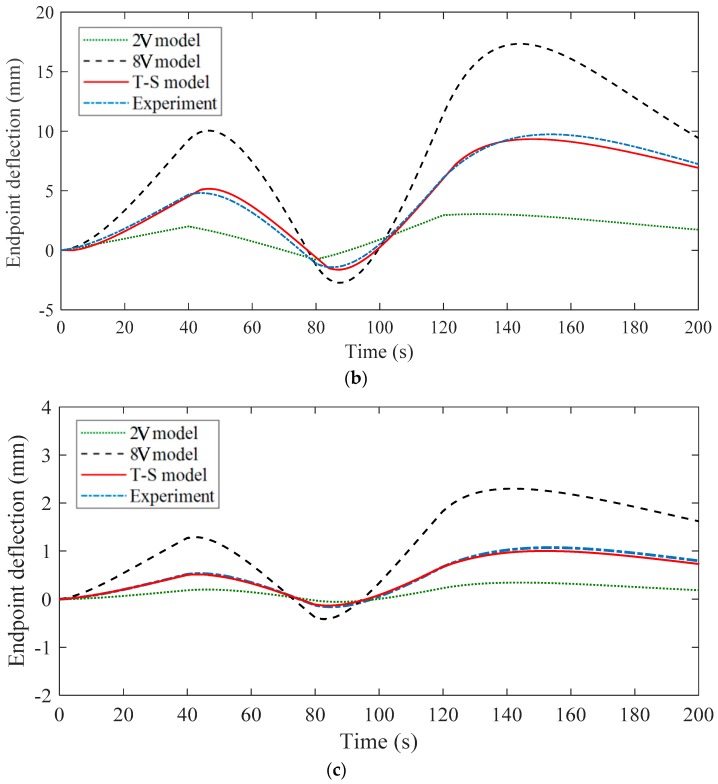
(**a**) Input voltage signal, (**b**) endpoint deflection of the arbitrary pattern actuator with size of S1, and (**c**) endpoint deflection of the lattice pattern actuator with size of S4.

A comparison of experimental tests with the T-S fuzzy model and estimated specific voltage models for 2 and 8 V input signals is shown in Figure 9 and Figure 10. The data is based on the average of three experiments to confirm reproducibility. Additionally, the efficacy of the developed model in terms of scalability of the 3D-printed soft actuators with different patterns and sizes are shown in Figure 10b,c where two actuators with arbitrary and lattice patterns and sizes S1 and S4 are compared in response to an analogous input. These figures show supremacy of actuator end-point position estimation by T-S fuzzy modelling compared to specific constant voltage models when changing the 3D-printed actuator parameters such as pattern and size. Furthermore, looking at Figure 10b in detail reveals some discrepancy of the T-S model from experimental results, especially over longer experimental tests. This is attributed to the time-varying intrinsic nature of the polyelectrolyte soft actuator that demands the feedback controller for a compensation purpose in future study.

## 5. Conclusions

A control-oriented modelling approach for 3D-printed polyelectrolyte soft actuators was presented in this study. First, a 3D-printed actuator with an arbitrary pattern was developed and characterized based on different sizes, electrolyte concentration, input magnitudes, and frequencies. Then, a linear transfer function of the 3D-printed polyelectrolyte soft actuator was developed to estimate the actuator behavior at different voltage signals. The T-S fuzzy model was further employed for a better presentation of the actuator model in a range of voltage variations by interrelating the voltage-dependent models. The experimental results showed improved performance obtained by using the T-S fuzzy model when compared to the linear transfer function at different voltages. The proposed model could be used for other 3D-printed soft actuators with custom design geometries due to its scalability.

## Figures and Tables

**Table 1:** The results of maximum endpoint deflection for the same pattern and different sizes of 3D-printed actuators.

Actuator	Length (mm)	Width (mm)	Thickness (mm)	Maximum Deflection (mm)
S1	40	8	2	5.92
S2	40	8	1	8.27
S3	40	4	2	5.38
S4	20	8	2	1.83
